# Improved Calculation Method of Coupling Factors for Low-Frequency Wireless Power Transfer Systems

**DOI:** 10.3390/ijerph19010044

**Published:** 2021-12-21

**Authors:** Jangyong Ahn, Seon-Eui Hong, Haerim Kim, Kyunghwan Song, Hyung-Do Choi, Seungyoung Ahn

**Affiliations:** 1 The CCS Graduate School of Green Transportation, Korea Advanced Institute of Science and Technology (KAIST), Daejeon 34051, Korea; jangyong.ahn@kaist.ac.kr (J.A.); haerim@kaist.ac.kr (H.K.); kyunghwan.song@kaist.ac.kr (K.S.); 2 Radio and Satellite Research Division, Electronics and Telecommunications Research Institute (ETRI), Daejeon 34129, Korea; sehong@etri.re.kr (S.-E.H.); choihd@etri.re.kr (H.-D.C.)

**Keywords:** electromagnetic field, exposure assessment, coupling factor, wireless power transfer

## Abstract

The concept of a coupling factor was introduced in International Electrotechnical Commission (IEC) 62311 and 62233 to provide a product safety assessment that considers the localized exposure when an electromagnetic field (EMF) source is close to the human body. To calculate the coupling factors between the human body and EMF source, a numerical calculation should be carried out to calculate the internal quantities of the human body models. However, at frequencies below 10 MHz, the computed current density or internal electric field has computational artifacts from segmentation or discretization errors. Specifically, coupling factors are calculated based on the maximum values, which may include computational artifacts due to abnormal peaks. In this study, we propose an improved calculation method to remove computational artifacts by applying the 99.99th percentile in calculating the coupling factors without underestimation. The performance of the proposed method is verified through a comparison based on various human body models with wireless power transfer (WPT) systems and compliance with the reference levels and basic restrictions. The results indicate that the proposed method can provide uniform coupling factors by reducing the computational errors by up to 65.3% compared to a conventional method.

## 1. Introduction

Wireless power transfer (WPT) technology has been continuously developed and is currently used in various applications. It has been studied in various categories, such as energy transmission using resonance in a low-frequency band or RF energy transmission, depending on the application [1,2,3]. In particular, in the low-frequency band, it has already been commercialized and widely used from relatively low-power wireless charging systems for phones to applications that use large size and high power, such as drones and electric vehicles [4]. As such, WPT technology is already being used to charge batteries to increase operating time and improve user convenience. In addition, as it is being studied steadily, its use is expected to increase further in the future.

However, advances in this technology have also led to concerns about the potential adverse effects on health caused by human exposure to electromagnetic fields (EMFs) from WPT systems. To protect the human body from EMFs radiated from the WPT systems, the international electrotechnical commission (IEC) technical report (TR) 62905 and IEC publicly available specification (PAS) 63184 defines the method for evaluating the EMF exposure of WPT systems in frequency bands below 10 MHz and 30 MHz, respectively [5,6]. In addition, international guidelines and standards recommend the limit of the leakage EMFs as a reference levels (RLs). However, since the RL is derived on the premise of a uniform exposure environment, an evaluation of the incident fields can be too conservative for an exposure environment in which EMFs are concentrated locally, such as WPT systems. Therefore, a coupling factor calculation method was proposed to compensate for the non-uniformity of the incident fields [7]. Although the evaluation of human exposure to EMFs from the WPT system is in progress as a standardized method, limitations still exist regarding the evaluation methods and results.

The assessment results of human exposure to EMFs have shown different results depending on the human body model even when evaluated for the identical WPT systems [8,9,10]. This is because different human body models have different dimensions, such as height, cross-section, and body shape, as well as different electrical properties of the tissues; thus, even if exposed to the equivalent magnetic field, the effects in the human body are different. In addition, at low frequency, computational artifacts from segmentation errors in anatomical models with imaging data or discretization errors in human body models with grid resolutions may occur [11,12,13]. Thus, the use of the 99th percentile of the current density (*J*) and internal electric field (*E*) was proposed and applied to reduce such artifacts and achieve a uniform assessment [14]. On the other hand, some studies argue that using the 99th percentile for non-uniform exposure may be insufficient because it can lead to underestimation [15,16,17]. Meanwhile, the coupling factor is calculated using the maximum value that can include artifacts, and this causes a significant difference in the coupling factor according to the human body model.

In this paper, we compare and analyze the magnetic field strength and induced quantities according to the human body models, WPT systems, and separation distance from human body models and systems. Based on these, we propose the use of 99.99th percentile for calculating a coupling factor at low frequency, which can objectively evaluate the exposure of the human body to EMFs by eliminating computational artifacts. With the proposed method, the coupling factors of each human body model are compared against the different WPT systems at different frequencies. In addition, by quantifying the maximum current of the system that avoids exceeding the limits, the proposed method is analyzed and verified through compliance testing under various magnetic field exposure environments.

## 2. Materials and Methods

### 2.1. WPT System Modeling

**Figure 1:**
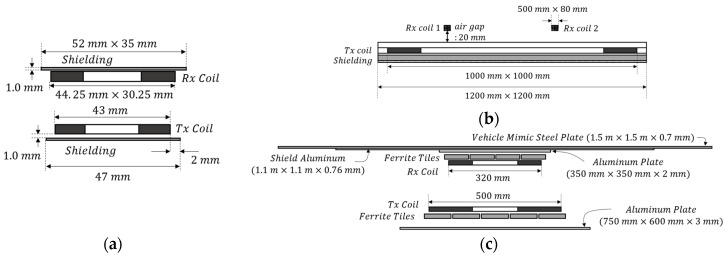
Geometry of WPT systems for (**a**) phones, (**b**) drones, and (**c**) electric vehicles.

Three representative WPT systems that are widely used are selected and modeled in consideration of their physical size and power level. Figure 1 illustrates the geometry of the three WPT systems considered in this study. The first is a 5 W class phone wireless charging system using a 111 kHz resonance frequency. Transmitting (TX) and receiving (RX) systems are designed based on TX A 10 and RX example 1 model, respectively, provided in Qi-standard [18]. The TX coil is a circular coil with an outer diameter of 43 mm, and the RX coil is a rectangular coil with an outer width of 44.25 mm × 30.25 mm. The modeling of the WPT systems for phones is satisfies the specifications in the standard. The second system is a WPT system for drones, operating at a resonance frequency of 140 kHz with a TX power of 200 W. The sizes of the TX and RX coils are 1000 mm × 1000 mm and 500 mm × 80 mm, respectively. The modeling of the system is confirmed by comparing electrical specifications and EMFs through simulations and experiments [10]. The last is an electric vehicle wireless charging system with a TX power of 7.7 kW. This is the WPT2/Z2 class circular system defined in the society of automotive engineers (SAE) standard, and it consists of a ground assembly and vehicle assembly corresponding to TX and RX, respectively, and a vehicle steel plate [19]. The TX and RX coils are quasi-square of 500 mm and 320 mm size, respectively, and, unlike other systems, 1.1 m × 1.1 m shield aluminum and 1.5 m × 1.5 m vehicle mimic steel plate are included. The modeling verifies that the design is accurate by satisfying the electrical specifications presented in the standard.

### 2.2. Human Body Models

**Figure 2:**
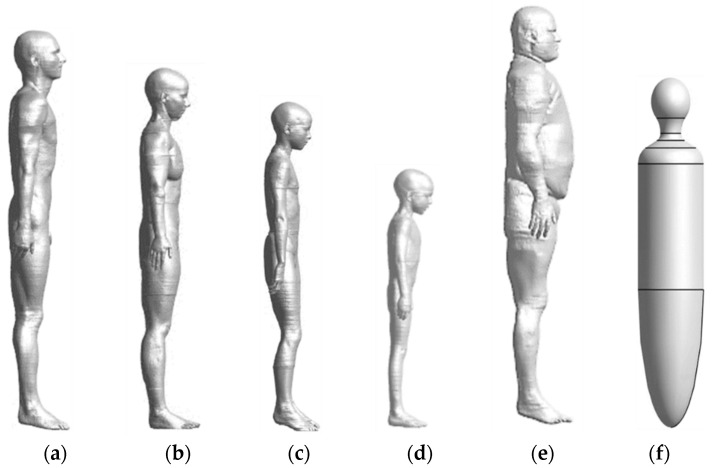
Anatomical human body models: (**a**) Duke, (**b**) Ella, (**c**) Billie, (**d**) Thelonious, (**e**) Fats, and simplified human body model: (**f**) uniform.

Five anatomical models and a simplified model, shown in Figure 2, are considered in this study. The five anatomical models are the virtual population models from information technologies in society (IT’IS) and the tissue properties of the IT’IS database are used [20,21,22]. The names and characteristics of each model are as follows: Duke (a 34-year-old male, with a body mass index (BMI) of 22.4 kg/m^2^), Ella (a 26-year-old female, with a BMI of 21.6 kg/m^2^), Billie (an 11-year-old female, with a BMI of 15.3 kg/m^2^), Thelonious (a 6-year-old male, with a BMI of 13.8 kg/m^2^), and Fats (a 37-year-old male, with a BMI of 36 kg/m^2^). A simplified model is a homogeneous human body model, introduced in the IEC standard, with two-thirds of the dielectric constant of the muscle at each operating frequency is used [23,24].

### 2.3. Computational Methods

#### 2.3.1. Coupling Factor

The method of evaluating human exposure to EMFs of the WPT system consists of four steps, the method using a coupling factor corresponds to tier 3. Tier 1 is a low-power exception condition, tier 2 is an incident EMFs evaluation against RLs, and tier 4 is internal quantities (*J*, *E*, and specific absorption rate (*SAR*)) evaluation against basic restrictions (BRs). The concept of a coupling factor is defined for non-uniform exposure conditions, i.e., a localized exposure scenario in which the human body is located within the vicinity of the WPT system [23,25]. The coupling factors can be calculated based on *J*, *E*, and *SAR*, respectively, and are denoted by *a_c_*, *a_c_*_1_, and *a_c_*_2_ in that order. Each calculation method is as shown in Equations (1)–(3) [5].

(1) $$a_{c}=\frac{J_{max}}{B_{max}\;}\times \;\frac{B_{lim}}{J_{lim}}$$

(2) $$a_{c1}=\frac{E_{max}}{H_{max}\;}\times \;\frac{H_{lim}}{E_{lim}}$$

(3) $$a_{c2}=\frac{\sqrt{SAR_{max}}}{H_{max}\;}\times \;\frac{H_{lim}}{\sqrt{SAR_{lim}}}$$

where *J_max_*, *E_max_*, and *SAR_max_* are the maximum values of the induced current density averaged over a 1 cm^2^ area, an electric field in a 2×2×2 mm^3^ cube, and a 10 g peak spatial-average SAR in the human body models. Here, *B_max_* and *H_max_* are the measured (or computed) spatial maximum field strength; *J_lim_*, *E_lim_*, and *SAR_lim_* are the values of the BRs; and *B_lim_* and *H_lim_* are the values of the RLs described in the guidelines [26,27]. Meanwhile, at a low frequency of below 10 MHz, the electrostimulation effects, such as the induced current density and electric field in the tissues are the dominant factors than the heating effects. As a result, only *a_c_* and *a_c_*_1_, which are based on nerve stimulation, are considered for coupling factors in this paper.

In the calculation process of *a_c_* and *a_c_*_1_, the difference between the two is *J_max_* and *E_max_*, which have significant differences in the numerical analysis process using complex human body models. *J_max_* is induced in the media of human tissue by Faraday’s law and is calculated by Equation (4). The current density is proportional to the frequency ω, the conductivity of the human body model σ, the current and number of turns of the TX/RX system *I* and *N*, and the mutual inductance between the loop formed by the eddy currents inside the human body model and the WPT system *M*. *M* is calculated through Equations (5) and (6), and the hypothetical loop radius of the current of the human body model, *R*, is determined as a value that maximizes *M* in the range less than the radius of the human body model [28].

(4) $$J=\frac{\sigma NM\omega I}{2\pi R}$$

(5) $$M=\mu_{0}\sqrt{R_{r}}\left[\left(\frac{2}{k}-k\right)F\left(k\right)-\frac{2}{k}E\left(k\right)\right]\;$$

(6) $$k=\frac{2\sqrt{R_{r}}}{\sqrt{{\left(R+r\right)}^{2}+d^{2}}}$$

Meanwhile, *E_max_* is calculated from vector potential *A* and scalar potential ϕ, which are based on Equations (7) and (8). In the process of being calculated, the electric field may introduce staircasing errors due to errors from developments in anatomical human body models. The reason is that the induced current rapidly changes direction in a sharp tissue structure such as the armpit of a human body model, generating a high electric field in the skin or the adjacent tissues [16].

(7) E=−jωA−∇ϕ

(8) $$A_{0}\left(\bar{r}\right)=\frac{\mu_{0}}{4\pi }\int^{\;}\frac{J_{0\left(\bar{r}\right)}}{\left|\bar{r}-{\bar{r}}^{\prime }\right|}d^{3}{\bar{r}}^{\prime }$$

The evaluation using the calculated coupling factor is finally compared with RL by multiplying the coupling factor by the incident EMFs. Since the method using the coupling factors is evaluated by compensating for non-uniformity in a local exposure condition, it is applied when the incident field does not satisfy RLs. In addition, even if non-uniformity is compensated by applying the coupling factor, it should be evaluated conservatively rather than evaluating internal quantities against BRs, because the BRs are based on established biological effects. The maximum permissible current (MPC) is used as a common indicator for each of these evaluation methods, which means the maximum allowable current applied to the system under the limit of each evaluation is reached. Thus, the MPC of tier 3 should be greater than that of tier 2 and smaller than that of tier 4.

#### 2.3.2. Numerical Methods

The modeling of the WPT systems and the calculations of human exposure to EMFs are conducted using a commercial electromagnetic simulation software Sim4Life version 5.1 [29]. Because the resonant frequencies of the WPT systems used in this research are less than 10 MHz, the magneto-quasi-static (MQS) approximation is applied [24]. The MQS method approximates a full-wave analysis based on the finite-difference-time-domain (FDTD) method, allowing the internal quantities to be calculated using the EMFs extracted from the WPT systems.

#### 2.3.3. Exposure Scenarios

The WPT systems for phones and drones are located at the center height of the human body models, which represents the worst-case exposure scenario when the system is changed to the vertical orientation [10]. The distances between the WPT system and the human body models vary from an extremely close distance of 10 mm to a distance at which the EMF strength is no lower than the RL. Because an assessment of human exposure using a coupling factor is not conducted at a distance at which the strength of the EMF is less than the RL. The coupling factors of the WPT systems for phones and drones are calculated within a distance of 30 mm and 100 mm, respectively, which are the effective distances of the coupling factor calculation method.

The drone and phone charging system are carried out at a certain height because the installation location is free, on the other hand, the WPT system for EV is set up on the ground because it is fixed on the ground. In addition, the WPT system for EV has an aluminum plate, shield aluminum, and a vehicle mimic steel plate on the upper part of the charging system, so that the leakage EMF is less than RL even if it is even a little away from the EV. Therefore, coupling factors are calculated only at one point right next to the EV, the only region exceeding RL.

## 3. Results

### 3.1. Comparison of Couping Factors and MPC Depending on the Human Body Models

**Figure 3:**
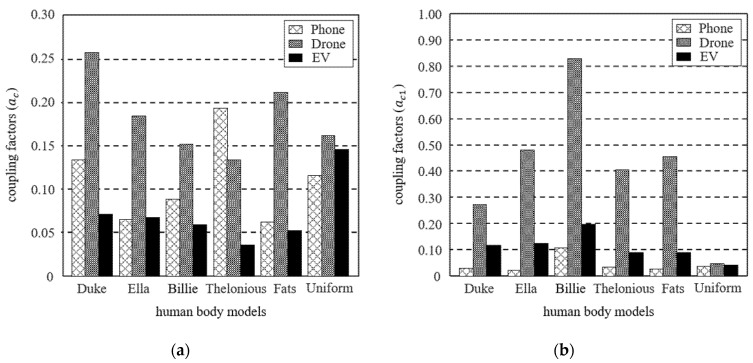
Comparison of coupling factors depending on the human body models of the three systems (**a**) *a_c_* and (**b**) *a_c_*_1_.

Figure 3 shows the coupling factors for each human body model of the three systems calculated at the nearest distance between the system and human body models. For both *a_c_* and *a_c1_* differ depending on the human body models. Even with the identical system, the difference depending on the human body model varies in terms of *M* and *R* in the exposed area in the case of *J*, and the difference in *E* is caused by the point where the maximum peak appears. Since the uniform human body model has a cross-sectional area similar to that of the anatomical human body models, the difference in *a_c_* is relatively small. On the other hand, *a_c_*_1_ of uniform human body model occurs much smaller than any other anatomical model because there is no valley such as armpits, fingers, and toes.

**Figure 4:**
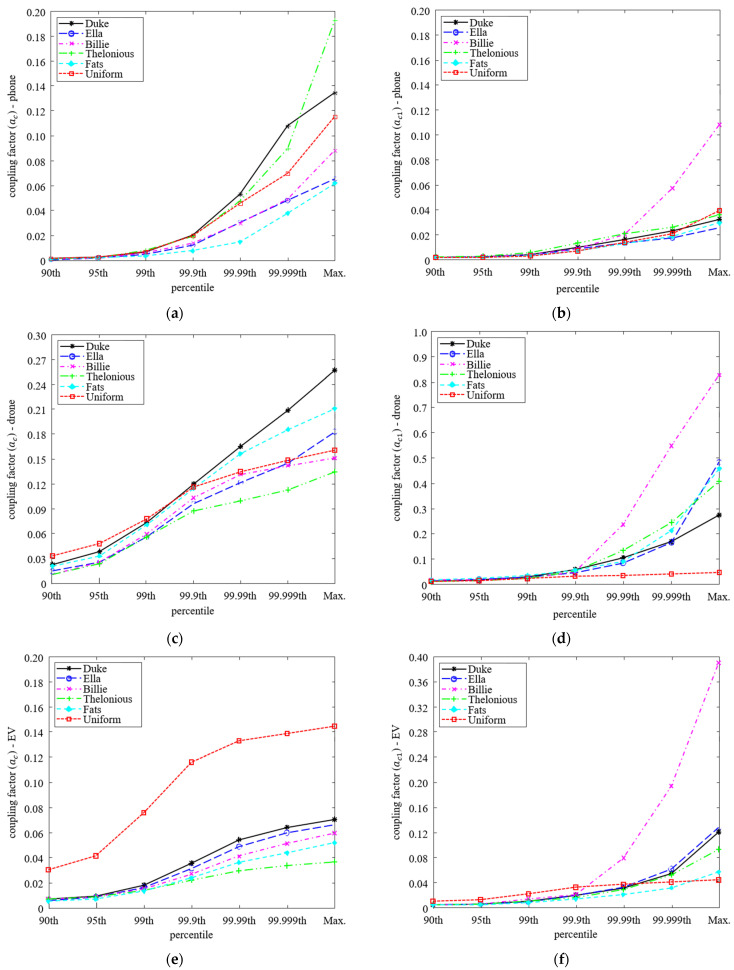
Comparison of coupling factors depending on percentile (**a**) *a_c,phone_*, (**b**) *a_c_*_1,*phone*_, (**c**) *a_c,drone_*, (**d**) *a_c_*_1,*drone*_, (**e**) *a_c,EV_*, and (**f**) *a_c_*_1,*EV*_.

Figure 4 shows the change in coupling factors depending on the percentile when *J* and *E* of each percentile are applied instead of *J_max_* and *E_max_* in (1) and (2), respectively. In the case of *a_c_*, all the human body models show a relatively similar tendency depending on the percentile in phone and drone systems, but the uniform model is calculated particularly large in the EV system. This is because the EV is installed on the ground, the *J_max_* occurs at the ankle of the anatomical models, which is smaller *R* compared to the torso of the uniform model. On the other hand, in the case of *a_c_*_1_, as *E_max_* appeared in the armpits and toes, a difference of up to about 4.4 times occurred between the anatomical models, and a difference of up to about 17.6 times occurred compared with the uniform model.

However, overall, the coupling factor decreases because the peak value decreases as the percentile decreases in all systems. In the tier 3 step of applying the coupling factor in the evaluation of human exposure to EMFs in the WPT system, the incident EMFs are multiplied by the coupling factor and compared with RL, so the reduction in the coupling factor is likely to be an underestimation.

On the other hand, when the percentile decreases, the difference between human body models also decreases. Reduction of the difference between human body models means that the results are not varied by the human body model, but uniform results are derived according to the characteristics of the system in evaluating the human exposure to EMFs of the system. To statistically analyze the variation between the models, the mean *m* and standard deviation σ of the coupling factors are calculated based on Equations (9) and (10).

(9) $$m=\frac{\sum_{i=1}^{N}a_{c}\left(i\right)}{N}=\frac{a_{c}\left(D\right)+a_{c}\left(E\right)+a_{c}\left(B\right)+a_{c}\left(T\right)+a_{c}\left(F\right)+a_{c}\left(U\right)}{N},$$

(10) $$\sigma =\sqrt{\frac{\sum_{i=1}^{N}{\left(a_{c}\left(i\right)-m\right)}^{2}}{N}}=\sqrt{\frac{{\left(a_{c}\left(D\right)-m\right)}^{2}+{\left(a_{c}\left(E\right)-m\right)}^{2}+{\left(a_{c}\left(B\right)-m\right)}^{2}+{\left(a_{c}\left(T\right)-m\right)}^{2}+{\left(a_{c}\left(F\right)-m\right)}^{2}+{\left(a_{c}\left(U\right)-m\right)}^{2}}{N}},$$

where *N* is the number of human body models and *D*, *E*, *B*, *T*, *F*, and *U* refer to Duke, Ella, Billie, Thelonious, Fats, and Uniform, respectively, which are the human body models shown in Figure 2.

Table 1 shows the *J* and *E*-based coupling factors and standard deviations of each three systems of phone, drone, and EV according to the percentile. As the percentile decreases in all systems, the standard deviations decrease, which means the difference between the human body models decreases. A reduction in percentile causes a decrease in abnormal peak in the human body model and a decrease in calculation uncertainty, so that uniform evaluation of the human body model can be conducted. However, even if it is possible to uniformly evaluate the human body model by reducing the calculation uncertainty and removing the abnormal peak appearing in a local area, there is a limit to reducing the percentile of the coupling factor. Therefore, it is necessary to select a percentile that has the minimum standard deviation and can be evaluated conservatively compared to tier 4 based on MPCs.

The MPCs of tier 3 are calculated by Equations (11), and tier 4 by (12) and (13). Here, by using Equations (1) and (2) into Equation (11), *MPC_tier3_* can be calculated by Equations (14) and (15). Here, *J_th_* and *E_th_* mean *J* and *E* values to which each percentile is applied like *J_99th_* or *E_99.999th_,* etc. Therefore, comparing Equations (12)–(15), respectively, it can be seen that the percentile of *MPC_tiers3_* is determined by the ratio of $H_{max\left(3cm^{2}\right)}$ (or $B_{max\left(3cm^{2}\right)}$) and $H_{max\left(100cm^{2}\right)}$. Since the ratio of $H_{max\left(3cm^{2}\right)}$ and $H_{max\left(100cm^{2}\right)}$ indicates how locally the magnetic field is concentrated at a specific point, it is determined according to the structure of the WPT system and the separation distance to the human body model. Thus, *MPC_tier3_* may vary according to the system characteristics and the separation distance between the system and the human body models.

(11) $$MPC_{tier3}=I_{\mathrm{system}}\times \frac{H_{lim}}{H_{max}\times a_{c}\left(or\;a_{c1}\right)}$$

(12) $$MPC_{tier4\left(J\right)}=I_{\mathrm{system}}\times \frac{J_{lim}}{J_{99th}}$$

(13) $$MPC_{tier4\left(E\right)}=I_{\mathrm{system}}\times \frac{E_{lim}}{E_{99th}}$$

(14) $$MPC_{tier3\left(J\right)}=I_{\mathrm{system}}\times \frac{H_{lim}}{H_{\max\left(100cm^{2}\right)}}\times \frac{B_{\max\left(3cm^{2}\right)}}{J_{th}}\times \frac{J_{lim}}{B_{lim}}$$

(15) $$MPC_{tier3\left(E\right)}=I_{\mathrm{system}}\times \frac{H_{lim}}{H_{max\left(100cm^{2}\right)}}\times \frac{H_{max\left(3cm^{2}\right)}}{E_{th}}\times \frac{E_{lim}}{H_{lim}}$$

### 3.2. Comparison of Couping Factors and MPC Depending on Distance

The coupling factor differs depending on the distance between the human body model and the WPT system even in the same system. Table 2 shows the coupling factor and the ratio of the H-field variation along with the distance. As the distance between the human body model and the WPT system increases, it becomes relatively close to the far-field, and thus the coupling factor also increases. On the other hand, the H-field ratio of 3 cm^2^ and 100 cm^2^ gradually decreases as the separation distance increases. This is because the average value of the magnetic field strength focused on a local area of 3 cm^2^ decreases as the separation distance increases.

One thing to note is that when all coupling factors in Table 2 are sorted in order of magnitude, *a_c_* is dominantly determined by the separation distance between the system and human body model, and *a_c_*_1_ is determined predominantly according to the type of system. As a result, *a_c_* is arranged in the order of separation distance rather than the system type, and *a_c_*_1_ is arranged in the order of phone, EV, drone, and in the order of distance within the system. This is because *J* is dominated by the magnetic field strength and *M* according to the distance, whereas in *E*, the peak is generated according to the system characteristics, and the exposed area is dominant. In addition, the H-field ratio is also in the order of phone, EV, and drone, which is the order of the radius size of the coil. The smaller the radius of the coil, the greater the magnetic field attenuation according to the distance, and the stronger magnetic field is concentrated in 3 cm^2^ because the magnetic field strength is strong at a close distance. This is the same reason why the phone, which has the smallest radius, has the greatest decrease in H-field ratio according to the distance compared to other applications.

**Figure 5:**
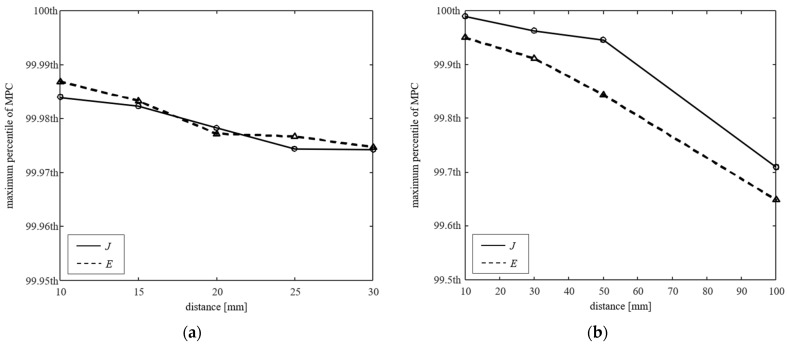
Maximum percentile of the MPCs according to the distance between the human body models and WPT systems (**a**) phone and (**b**) drone.

Figure 5 shows the maximum percentile of the MPCs according to the distance of the phone and drone systems. This is the maximum percentile that is not estimated to be less than *MPC_tier_*_4_ when applied to *J_th_* or *E_th_* in Equations (14) and (15), respectively. As the distance increases, the MPCs also decreases, because the H-field ratio decreases. When the system is close to the human body, it may be underestimation rather than tier 4 if peaks are removed under 99.99th percentile, and further peaks can be removed as the system is farther away.

**Figure 6:**
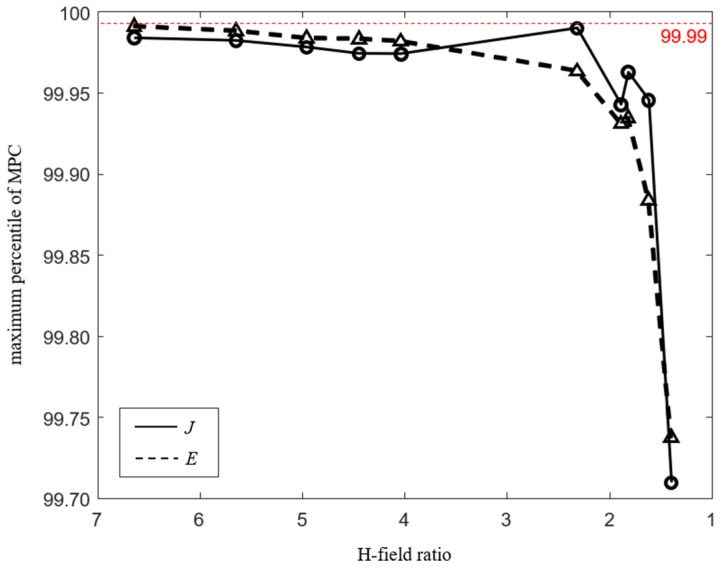
MPCtier3 according to H-field ratio.

Figure 6 shows the aggregated results of the maximum percentile of MPCs according to the H-field ratio for all systems. Since the H-field ratio increases as the distance between the WPT system and the human body model decreases, the x-axis is shown in the direction in which the distance increases, that is, in which the H-field ratio decreases. As the distance increases, the maximum percentile also decreases according to the decreasing H-field ratio. The 99.99th percentile is suitable for conservative evaluation regardless of the distance, and the percentile can be determined according to the H-field ratio for the uniform assessment regardless of the human body model.

**Figure 7:**
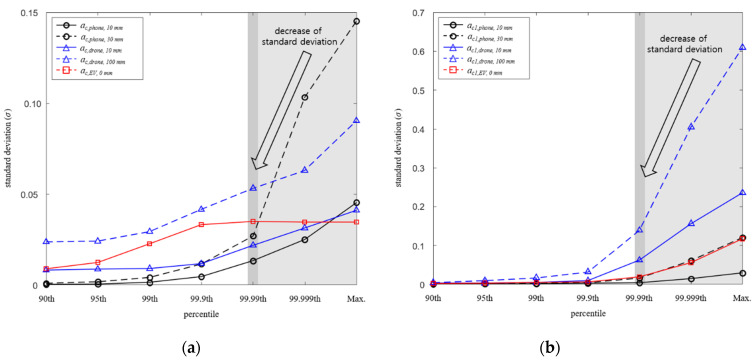
Standard deviations depending on the percentiles. (**a**) *a_c_* and (**b**) *a_c_*_1_.

Figure 7 shows the standard deviation depending on the percentiles for each system. As the percentile decreases, the variation between the human body models decreases, so that the σ decreases. As described in the analysis of coupling factor reduction, the decrease in $\sigma_{c1}$ was up to 88.8%, which is higher than that of $\sigma_{c}$ of 81.5% maximum. However, as shown in Figure 4e, due to the change in coupling factor difference between the uniform model and anatomical models according to percentile remaining almost constant, the $\sigma_{c,\mathrm{EV}}$ change according to percentile hardly appears.

At the maximum distance that does not exceed RL, the conventional calculation method shows the differences between the human body models as 3.5-, 1.9-, and 4-times for *a_c_*, and 7.8-, 19.5-, and 6.5-times for *a_c_*_1_ in phone, drone, and EV systems, respectively. On the other hand, the differences between the human body models of applying 99.99th percentile are 2.8-, 1.6-, 4-times for *a_c_*, which is reduced by up to 19.5% and 2.7-, 6.8-, 4.2-times for *a_c_*_1_, which is reduced up to 65.3%. As a result, applying the 99.99th percentile instead of the maximum value achieves the uniform assessment without an underestimation in comparison to the tier 4 evaluation.

## 4. Conclusions

In this paper, we proposed an improved coupling factor calculation method for removing computational artifacts to achieve uniform assessment results in human body models exposed to EMFs from WPT systems. Three different WPT systems (phone, drone, and EV) are modeled to calculate the internal quantities and coupling factors in the human body models. The coupling factors are then calculated and compared with five anatomical models and a simplified model. The coupling factor *a_c_*, which is calculated based on the current density, shows a difference of 3.1-, 1.9- and 4.0-times the minimum and maximum values of six human body models in the three WPT systems. The coupling factor *a_c_*_1_, which is calculated based on the internal electric field, has a difference of 4.4-, 17.6-, and 6.5-times the values. To achieve a uniform coupling factor by removing the computational artifacts, we compared the results according to the percentiles and suggested using the 99.99th percentile, which can be the most generalized value without an underestimation against the tier4 evaluation. When the proposed method is applied, the errors in *a_c_* and *a_c_*_1_ are reduced by up to 19.5% and 65.3%, respectively, compared to that of the conventional method. As a result, by applying the proposed method, a relatively uniform coupling factor can be calculated even if a specific human body model is evaluated without having to evaluate all other human body models. Moreover, there is a possibility to further reduce the errors by applying the lower percentile according to the H-field ratio.

## Figures and Tables

**Table 1:** Coupling factors according to percentiles and corresponding standard deviations.

		Percentile						
		90th	95th	99th	99.9th	99.99th	99.999th	Max
Phone	$a_{c,phone}$	0.001	0.002	0.006	0.015	0.037	0.067	0.110
	$\sigma_{c,phone}$	0.0003	0.0003	0.0013	0.0044	0.0132	0.0249	0.0453
	$a_{c1,phone}$	0.0007	0.0011	0.0029	0.0079	0.0153	0.0263	0.0444
	$\sigma_{c1,phone}$	0.0002	0.0003	0.0009	0.0022	0.0032	0.0138	0.0287
Drone	$a_{c,drone}$	0.019	0.032	0.066	0.106	0.135	0.157	0.183
	$\sigma_{c,drone}$	0.008	0.009	0.009	0.012	0.022	0.031	0.041
	$a_{c1,drone}$	0.014	0.018	0.028	0.049	0.115	0.231	0.416
	$\sigma_{c1,drone}$	0.003	0.003	0.005	0.009	0.062	0.155	0.235
EV	$a_{c,EV}$	0.010	0.014	0.026	0.043	0.057	0.065	0.072
	$\sigma_{c,EV}$	0.009	0.012	0.023	0.033	0.035	0.035	0.035
	$a_{c1,EV}$	0.003	0.005	0.010	0.018	0.036	0.071	0.137
	$\sigma_{c1,EV}$	0.002	0.003	0.005	0.006	0.019	0.056	0.117

**Table 2:** Coupling factor and the H-field ratio depending on the distance.

**System**	**Phone**				
Distance	10 mm	15 mm	20 mm	25 mm	30 mm
$a_{c}$	0.110	0.154	0.202	0.256	0.315
$a_{c1}$	0.044	0.058	0.075	0.097	0.118
$H_{max\left(3cm^{2}\right)}/H_{max\left(100cm^{2}\right)}\;$	6.64	5.65	4.96	4.45	4.04
**System**	**Drone**				**EV**
Distance	10 mm	30 mm	50 mm	100 mm	0 mm
$a_{c}$	0.183	0.275	0.326	0.392	0.072
$a_{c1}$	0.416	0.672	0.845	1.116	0.137
$H_{max\left(3cm^{2}\right)}/H_{max\left(100cm^{2}\right)}\;$	2.32	1.82	1.62	1.40	1.89

## Data Availability

The data within this systematic review and is available through the relevant articles referenced throughout.
